# A Repertory to the Cyclopædia of Drug Pathogenesy

**Published:** 1897-09

**Authors:** 


A Repertory to the Cyclopædia of Drug Pathogenesy. Compiled by Richard Hughes, M. D. Part I, containing Introduction, Nervous System, and Head. London: E. Gould & Son, 59 Moorgate Street, E. C. New York: Boericke & Tafel, 159 Grand St. 1897.

This book, as its name indicates, is a repertory to the well-known *Cyclopædia of Drug Pathogenesy*, which was published some years ago with incredible labor and great discouragement by our English cousins.

This *Cyclopædia*, notwithstanding its name, does not contain Hahnemann's own contributions to materia medica, the reason for this being that as they could not be improved upon as already published, they would have to be transferred bodily to the *Cyclopædia*, which would be a needless repetition, and greatly increase the bulk and the cost of the *Cyclopædia*. The special matter of the *Cyclopædia* is therefore merely a *supplement* to Hahnemann's work, in the *Materia Medica Pura*. Consequently this *Repertory*, now under review, is an index not alone to the volumes of the *Cyclopædia*, but also the three works of Hahnemann, entitled *Materia Medica Pura*, *Chronic Diseases*, and *Fragmenta de Viribus*. It should be understood that the edition of the *Chronic Diseases* referred to is the new translation of 1896 by Professor Louis H. Tafel, reviewed in The Homœopathic Physician for April, 1896, page 195.

The fact that the book under notice is a repertory to Hahnemann's *Materia Medica*, and to this new edition of *The Chronic Diseases*, as well as to their supplement, the *Cyclopædia*, will at once arrest attention and excite interest in it, far beyond what would be the case were it recognized simply as a *Repertory* of the *Cyclopædia*.

The *Cyclopædia* has had a hard road to travel in getting the patronage of the profession from five principal causes. The first of these was an opposition developed by the bitter attacks of many of its partisans upon the authenticity of other works of materia medica; the calling in question of the whole materia medica of the school, and an avowed determination to break it down and destroy it. In the introduction we find two or three sentences which will be recognized by older practitioners as the key-note of the division of the school into two principal factions, and as the bottom of the attacks on the materia medica. These sentences we quote: "Those who use it believe that the best medicine for a given morbid condition is one which has shown the power of inducing its *simile* on the healthy subject. They are, as students meditating on the types of disease described in books, or as practitioners, confronting the varieties of the same encountered in daily practice."

The second cause was the commonly accepted belief that the builders of the *Cyclopædia* had actually thrown out valuable symptoms on one pretext or another, all in line with the above-stated questioning of the whole fabric of materia medica and the mode of thought indicated by the quoted sentences.

The third cause was that we were, especially in America, pretty well supplied with Dr. T. F. Allen's *Encyclopædia of Pure Materia Medica* and Hering's *Guiding Symptoms*, and hardly felt the need of another such work.

The fourth cause was the solid way in which the type was set up in the *Cyclopædia*, which made it look a hopeless task for the seeker after any particular symptom, and the fifth cause was the absence of any key to unlock these intricacies.

The first of these causes has about died out. Students of materia medica realized that while they had their own printed copies of the *Materia Medica*, and were able to make cures from them, and so hold their confidence in themselves and their means of treatment, and could also secure the confidence and gratitude of their patients, the materia medica was in no danger of being lost from any attacks made upon it, however virulent and mistaken.

The second cause has also died out, since it has been realized that if certain symptoms were expurgated, those who brought about the expurgation were at least seeking the truth on their own account. The third cause also no longer counts, as every sensible student knows that in every department of learning not all the knowledge is contained in the works of one author, and that we cannot have too much assistance in the difficult task of finding the simillimum. The fourth objection will be largely obviated on the completion of this *Repertory*, which thus effectually disposes of the fifth objection.

Now as to the arrangement of the *Repertory*, "the first step in the present indexing has been to go through the provings, poisonings, and experiments on animals, pen in hand, noting the phenomena to which reference may be made. It is granted that much judgment is required for such a process, and that some risk has been run. But this is better than rendering the *Repertory* unwieldy and untrustworthy by filling it with sign posts which point to quicksands. Nothing has been omitted which has anything distinctive about it, whether it be in substantiva or in adjectiva. It is simply that our whole symptomatology has been dealt with for indexing purposes as Hahnemann dealt with Nenning's contributions thereto, extracting only that which seemed really useful."

The foregoing paragraph inclosed in quotation marks is copied from the Introduction. Otherwise it may be said that the *Repertory* is not arranged on the alphabetical but rather upon the schematic plan. Some new anatomic regions are created in accordance with the author's views of how the grouping should be made, and then the symptoms are detailed in double column and the names of the remedies given much abbreviated. The whole is preceded by a table of these abbreviations, so that they may be readily understood.

The reader should distinctly understand that the book under discussion is not a completed work, but only the beginning or first fascicle of the *Repertory*. It is a pamphlet of ninety-six pages, in paper cover, and the remaining sections are to be issued as fast as possible. No doubt a liberal subscription by the profession will hasten the work, and we hope that this it will get.

Of the price we are not advised, but as soon as we learn it our readers will be duly informed.

